# Prevalence of excessive body fat among adolescents of a south Brazilian metropolitan region and State capital, associated risk factors, and consequences

**DOI:** 10.1186/s12889-018-5216-0

**Published:** 2018-03-02

**Authors:** Leandra Ulbricht, Mariane Ferreira de Campos, Eduardo Esmanhoto, Wagner Luis Ripka

**Affiliations:** Graduate Program in Biomedical Engineering, Federal University of Technology – Paraná, Av. Sete de Setembro, 3165 – Rebouças – CEP, Curitiba, PR 80230-901 Brazil

**Keywords:** Excessive body fat, Prevalence and risk factors, Adolescents

## Abstract

**Background:**

The prevalence of overweight/obesity has become a major concern for public health in developing countries. Risk factors need to be well documented so that these countries develop public policies to fight the problem. Thus, the objective of this study is to determine the prevalence of excess fat among adolescents of a South Brazilian State Capital associated with risk factors and their consequences.

**Methods:**

This study was conducted between 2014 and 2016 with adolescents aged 11–18 years. The following body composition measurements were collected: body mass, height, waist circumference, fat mass and bone mineral density (this latter through dual energy X-ray absorptiometry). Biochemical data as glucose, total cholesterol, and triglycerides were also collected. Finally, socioeconomic questionnaires were applied, as well as questionnaires regarding: the education level of guardians, active transportation, time spent with sedentary activities and physical activities. Odds ratios and chi-square test were applied in statistical analysis.

**Results:**

Data from 675 adolescents, from which 70% were males, were analyzed. The mean age was 14.7 ± 1.8 years. The prevalence of excess fat was 18.2% in boys and 92.1% in girls. As for sedentary lifestyle and physical inactivity, having one of these factors increased the risk of being overweight by 7.9 times for boys and 3.0 times for girls. In boys, there was a significant association between excess fat and waist circumference (*p* = 0.000; OR = 13.5; CI = 7.0–25.9), physical activity level (*p* = 0.000; OR = 4.0; CI = 2.5–6.5), triglycerides (*p* = 0.019; OR = 2.2; CI = 1.1–4.2) and total cholesterol (p = 0.000; OR = 2.6; CI = 1.6–4.5). In girls, there was an association between having excess fat and an increase in total cholesterol (p = 0.000; OR = 8.0; CI = 2.6–24.4).

**Conclusions:**

The high prevalence of excess fat was greater than what was described by some studies conducted in developed countries. This reality demonstrates the need to implement public policies that can directly promote the reduction of sedentary habits and reinforce the importance of adopting an active lifestyle.

**Electronic supplementary material:**

The online version of this article (10.1186/s12889-018-5216-0) contains supplementary material, which is available to authorized users.

## Background

Non-communicable diseases can originate during childhood and adolescence, and they are currently the leading cause of mortality and morbidity in most populations [1, 2]. The current epidemic of overweight and obesity [3, 4] fits this description. In Latin America, the rising rate of overweight and obesity observed in the last three decades has not spared children nor adolescents. Data from 2014 indicate that this prevalence may represent 25% (51.8 million) of the total population of children and adolescents in the region [3].

As motivation for this study, the literature presents different prevalence results for overweight and obesity among children and adolescents in developing countries. This situation raises the hypothesis that in this phenomenon there are sociodemographic and lifestyle differences that can have impact on the different risk factors [3, 5]. Brazilian studies have no different conclusions and maintain this discrepancy, with rates ranging from 4.4% to 18% for obesity [6] and 3.1% to 38.9% for overweight [7]. This variation is due to the instruments used in the measurements, the methodology used for the cut-off point and the regions of the country being studied [6, 7].

Thus, the relationship between obesity and a number of variables such as socioeconomic status, sex, age, sedentary habits, education level of guardians, active transportation, among others, can be better documented [6, 8, 9].

In order to fight the problem of excess fat, the causes and risk factors must be known. The main diseases associated with overweight and obesity are: hypertension, heart disease, type 2 diabetes mellitus, hypercholesterolemia, gall bladder diseases, obstructive sleep apnea, non-alcoholic steatohepatitis, mental health concerns, asthma, osteoporosis, negative impact on quality of life, etc. [2–4, 6, 8, 10, 11].

The excess fat in young people that was once a challenge to the public health of developed countries now represents a growing threat to developing countries [2, 9]. However, although 80% of the world’s population is located in these countries, only a small fraction of the researches focusing on its determinants or risk factors are being performed in these countries [12, 13]. Given that Brazil is a continental size country, the development of research in different regions may be fundamental to learn the reality of public health.

As follows, considering: a) that the association between excess fat, socio-demographic factors, and lifestyle in developing countries is not yet clear; b) the serious consequences that can result from excess fat; and c) the need for regional studies to establish more effective local preventive measures; the aim of this study is to determine the prevalence of excess fat among adolescents from a south Brazilian metropolitan region and State capital, associated with risk factors and their consequences.

## Methods

### Study population

This is a cross-sectional study carried out from 2014 to 2016, with adolescents (11–18 years) whose parents gave permission to participate in the research by signing an informed consent form. For the sample size, a sampling error of 4.5% was specified at 91% confidence level of a universe of 82,414 individuals (Additional file 1). The study sample consisted of adolescents who met the following criteria: a) parents authorized their participation; b) did not make use of medicines containing calcium; c) did not undergo radiography/computed tomography in the seven days prior to the evaluation; d) did not suspect pregnancy. The subjects of the study were gathered from public and private schools, as well as sports training centers in the metropolitan region (composed of the city of Curitiba-PR, Brazil, and 29 other municipalities).

The work was ethically approved through the *Plataforma Brasil* system under the protocol No. 11583113.7.0000.5547.

### Anthropometric and biochemical evaluation

The body mass was assessed with a mechanical scale and the height was assessed using a stadiometer coupled to the scale (Filizola, São Paulo, Brazil). The waist circumference was measured in the smallest circumference of the abdomen with a flexible and inelastic tape measure (Cardiomed, Curitiba, Brazil). For classification purposes, the adopted cut-off point was ≥80 cm for boys and ≥90 cm for girls [14].

The evaluation of body fat and bone mineral density was performed using dual energy X-ray absorptiometry (DXA) technology using a Hologic Discovery A linear sweep fan-beam scanner *(*Hologic, Inc., Bedford, U.S.A.). All DXA procedures were performed by an expert in the equipment. For the classification of the excess fat, the cut-off point of Williams et al. was used, which establishes a percentage of fat (%BF) with a value equal to or greater than 25% for boys, and 30% for girls [15]. With regard to the BMI classification, it was made from the index calculation (kg/m^2^) using the Brazilian recommendation of Conde and Monteiro [16].

Serum glucose, total cholesterol (TC) and triglyceride levels were measured using a portable test-strip type dosing equipment (Accutrend Plus, Roche, Germany). For the classification of the individuals, the reference values of the Brazilian Society of Diabetes were used, being classified as individuals with high levels those with glucose levels ≥140 mg/dl, and triglycerides levels ≥150 mg/dl. For TC, 150 to 169 mg/dl were considered as limit values, and values ≥170 mg/dl were considered as high values.

### Evaluation of physical activity, sedentary lifestyle and socioeconomic profile

The levels of physical activity and sedentary lifestyle were evaluated through the International Physical Activity Questionnaire (IPAQ) short version, validated in Brazil by Matsudo et al. [17]. The IPAQ is a questionnaire that allows estimating the weekly time spent in moderate and vigorous physical activities in different contexts of everyday life, such as work, transportation, domestic tasks and leisure, as well as the time spent in passive activities performed in the sitting position.

As classification criteria for the level of physical activity, the guidelines of the World Health Organization were followed*.* Adolescents who accumulated at least 60 min of moderate or vigorous physical activity per day were labeled as active. Sedentary habits (time spent on television, computer and video games) were calculated separately for weekdays and weekends. They were categorized as excessive if the value was greater than 2 h a day.

A classification of aggregated factors was proposed, in which the individuals were grouped into three categories: a) adequate physical activity and not sedentary (classified as “no factor”); b) either no physical activity or sedentary (classified as “1 factor”); c) low levels of physical activity and sedentary (classified as “2 factors”).

The socioeconomic profile was assessed through the questionnaire proposed by the Brazilian Association of Research Companies (ABEP in the Portuguese abbreviation). This instrument allows estimating the purchasing power of families and classifying them into classes: A1, A2, B1, B2, C1, C2, D and E. For this, the subjects were asked to answer nine questions about how many of these items/services they had at home: TV, refrigerator, monthly house cleaner, automobile, bathroom and DVD player. The classes were gathered into three categories: A1 and A2 (high purchasing power category), B1 and B2 (medium purchasing power category) and C1, C2, D and E (low purchasing power category) [18].

### Statistical procedures

For the statistical analysis, the sample was characterized with position (mean) and dispersion (standard deviation) measurements, as well as frequency distribution (%). To investigate the association between excess fat and factors such as biochemical, socioeconomic, active transportation, physical activity level and sedentary lifestyle, the chi-square test was applied. The risk ratio [odds ratio (OR)] and its 95% confidence intervals (95% CI) were obtained through a specific procedure of the *Statistical Package for the Social Sciences* (SPSS), version 17.0 (SPSS Inc. Chicago, IL). The value of *p* < 0.05 was adopted as statistical significance.

## Results

Data from 675 adolescents, from which 70% were males, were analyzed. The mean age and mean fat percentage were 14.7 ± 1.8 years and 20.9 ± 5.9% for boys, 14.5 ± 2.0 years and 33.0 ± 5.4% for girls, respectively (Table 1).

**Table 1:** Descriptive analysis of data derived from excess fat, sedentary lifestyle, physical activity and biochemical markers

Variable	Boys	Girls	All
N	472	203	675
Age (years)	14.7 ± 1.8	14.5 ± 2.0	14.7 ± 1.8
Weight (kg)	59.9 ± 13.5	55.9 ± 11.5	58.7 ± 13.1
Height (m)	1.67 ± 0.1	1.59 ± 0.1	1.65 ± 0.1
BMI (kg/m^2^)	21.3 ± 4.3	22,3 ± 4.06	21.6 ± 4.2
WC (cm)	70.9 ± 8.6	68.8 ± 7.9	70.3 ± 8.4
Fat Mass (%)	20.9 ± 5.9	33.0 ± 5.4	24.5 ± 8.0
Bone Mineral Density (g/cm^2^)	1.064 ± 0.137	0.995 ± 0.112	1.04 ± 0.133
%BF Classification			
Normal	386 (81.8%)	16 (7.9%)	402 (59.6%)
High	86 (18.2%)	187 (92.1%)	273 (40.4%)
BMI Classification			
Normal Weight	330 (69.9%)	121 (59.6%)	451 (66.8%)
Overweight/Obesity	142 (30.1%)	82 (40.4%)	224 (33.2%)
Biochemical Classification			
Cholesterol Normal	224 (47.5%)	50 (24.6%)	274 (40.6%)
Cholesterol Limit (150–169 mg/dl)	118 (25.0%)	61 (30.0%)	179 (26.5%)
Cholesterol High (≥170 mg/dl)	105 (22.2%)	86 (42.3%)	191 (28.3%)
Cholesterol (unrealized)	25 (5.3%)	6 (3.0%)	31 (4.6%)
Blood Glucose Normal	445 (94.3%)	192 (94.6%)	637 (94.4%)
Blood Glucose High (≥140 mg/dl)	4 (0.8%)	1 (0.5%)	5 (0.7%)
Blood Glucose (unrealized)	23 (4.9%)	10 (4.9%)	33 (4.9%)
Triglycerides Normal	395 (83.7%)	163 (80.3%)	558 (82.7%)
Triglycerides High (≥150 mg/dl)	50 (10.6%)	34 (16.7%)	84 (12.4%)
Triglycerides (unrealized)	27 (5.7%)	6 (3.0%)	33 (4.9%)
Physical activity level			
Time (hours/day)	2.2 ± 2.0	1.7 ± 2.1	2.0 ± 2.0
Active	313 (66.3%)	106 (52.2%)	419 (62.1%)
Inactive	159 (33.7%)	97(47.8%)	256 (37.9%)
Sedentary lifestyle			
Time in week (hours/day)^a^	4.3 ± 3.5	4.6 ± 3.4	4.4 ± 3.4
Time in weekend (hours/day)^a^	6.5 ± 5.9	5.9 ± 4.6	6.2 ± 5.3
% of sedentary	355 (75.2%)	161 (79.3%)	545 (80.8%)
Socioeconomic profile			
High	21 (4.4%)	8 (3.9%)	29 (4.3%)
Medium	170 (36.0%)	78 (38.4%)	248 (36.7%)
Low	194 (41.1%)	50 (24.6%)	244 (36.1%)
Not answered	87 (18.5%)	67 (33.1%)	154 (22.9%)
Active transportation			
Yes	154 (32.6%)	64 (31.6%)	218 (32.3%)
No	174 (36.8%)	115 (56.6%)	289 (42.8%)
Not answered	144 (30.6%)	24 (11.8%)	168 (24.9%)

Where: waist circumference (WC); body mass index (BMI); body fat percentage (%BF); physical activity (PA); time spent with sedentary habits (^a^);

The prevalence on excess fat was 40.4% (18.2% of boys and 92.1% of girls). Regarding the biochemical parameters, cholesterol levels were the most altered in both sexes, even though the excess fat was much more prevalent in females. If we add adolescents with total cholesterol levels at the limit and higher, we have 370 adolescents (54.8%) with altered levels. An additional analysis concerning different age groups was presented in the Additional file 2.

Although 66.3% of the boys and 52.2% of the girls met the WHO criteria for classification of active individuals, 75.2% of the boys and 79.3% of the girls also had sedentary habits, mainly on weekends, reaching a mean of 6.5 ± 5.9 h per day for boys and 5.9 ± 4.6 h per day for girls.

The relations between excess fat and the waist circumference, physical activity level, sedentary habits, blood glucose levels, socioeconomic data, and active transportation, were presented for all group in Table 2, and then subdivided into boys and girls in Tables 3 and 4, respectively.

**Table 2:** Distribution of excess fat according to indicators of physical activity, sedentary lifestyle, socioeconomic data, active transportation and biochemical data for all group

Variables	Condition	Excess body fat		*P*	Odds ratio (95% CI)
		No	Yes		
Waist Circumference (*n* = 675)	Normal	385 (57.0%)	237 (35.1%)	0.000	3.4 (1.89–6.3)
	Elevated	17 (2.5%)	36 (5.3%)		
Physical Activity (*n* = 675)	Active	290 (43.0%)	129 (19.1%)	0.000	2.9 (2.1–4.0)
	Inactive	112 (16.6%)	144 (21.3%)		
Sedentary Lifestyle (*n* = 675)	Yes	303 (44.9%)	213 (31.6%)	0.426	–
	No	99 (14.7%)	60 (8.9%)		
Aggregate factors (*n* = 675)^a^	No Factor	45 (6.7%)	16 (2.4%)	0.000	1.5 (0.81–2.7)
	1 Factor	299 (44.3%)	157 (23.3%)		
	2 Factors	58 (8.6%)	100 (14.8%)		4.8 (2.5–9.1)
Active Transportation (*n* = 507)	Yes	127 (25.0%)	91 (17.9%)	0.233	–
	No	153 (30.2%)	136 (26.8%)		
Socioeconomic profile (*n* = 521)	Low	165 (24.4%)	79 (11.7%)	0.299	–
	Medium	143 (21.2%)	105 (15.6%)		
	High	22 (3.3%)	7 (1.0%)		
Triglycerides (*n* = 642)	Normal	345 (53.7%)	213 (33.2%)	0.001	2.2 (1.4–3.4)
	High	36 (5.6%)	48 (7.5%)		
Cholesterol Total (*n* = 644)	Normal	210 (32.6%)	64 (9.9%)	0.000	3.8 (2.7–5.3)
	High	172 (26.7%)	198 (30.7%)		
Blood Glucose (*n* = 642)	Normal	380 (59.2%)	257 (40.0%)	0.355	–
	High	4 (0.6%)	1 (0.2%)		

^a^Aggregate factors: a) adequate physical activity and not sedentary (classified as “no factor”); b) either no physical activity or sedentary (classified as “1 factor”); c) low levels of physical activity and sedentary (classified as “2 factors”)

**Table 3:** Distribution of excess fat according to indicators of physical activity, sedentary lifestyle, socioeconomic data, active transportation and biochemical data for boys

Variables	Condition	Excess body fat		*P*	Odds ratio (95% CI)
		No	Yes		
Waist Circumference (*n* = 472)	Normal	369 (78.2%)	53 (11.2%)	0.000	13.5 (7.0–25.9)
	Elevated	17 (3.6%)	33 (7.0%)		
Physical Activity (*n* = 472)	Active	279 (59.1%)	34 (7.2%)	0.000	4.0 (2.5–6.5)
	Inactive	107 (22.7%)	52 (11.0%)		
Sedentary Lifestyle (*n* = 472)	Yes	293 (62.1%)	62 (13.1%)	0.459	–
	No	93 (19.7%)	24 (5.1%)		
Aggregate factors^a^ (*n* = 472)	No Factor	41 (8.7%)	1 (0.2%)	0.000	7.9 (1.1–58.7)
	1 Factor	290 (61.4%)	56 (11.9%)		
	2 Factors	55 (11.7%)	29 (6.1%)		11.3 (2.6–50.2)
Active Transportation (*n* = 328)	Yes	124 (37.8%)	30 (9.1%)	0.801	–
	No	142 (43.3%)	32 (9.8%)		
Socioeconomic profile (*n* = 385)	Low	161 (34.1%)	33 (7.0%)	0.299	–
	Medium	137 (29.0%)	33 (7.0%)		
	High	20 (4.2%)	1 (0.2%)		
Triglycerides (*n* = 445)	Normal	330 (74.2%)	65 (14.6%)	0.019	2.2 (1.1–4.2)
	High	35 (7.9%)	15 (3.4%)		
Cholesterol Total (*n* = 447)	Normal	199 (44.5%)	25 (5.6%)	0.000	2.6 (1.6–4.5)
	High	167 (37.4%)	56 (12.5%)		
Blood Glucose (*n* = 449)	Normal	364 (81.1%)	81 (18.0%)	0.346	–
	High	4 (0.9%)	0 (0%)		

^a^Aggregate factors: a) adequate physical activity and not sedentary (classified as “no factor”); b) either no physical activity or sedentary (classified as “1 factor”); c) low levels of physical activity and sedentary (classified as “2 factors”)

**Table 4:** Distribution of excess fat according to indicators of physical activity, sedentary lifestyle, socioeconomic data, active transportation and biochemical data for females

Variables	Condition	Excess body fat		*P*	Odds ratio (95% CI)
		No	Yes		
Waist Circumference(*n* = 203)	Normal	16 (7.9%)	184 (90.6%)	0.610	–
	Elevated	0 (0%)	3 (1.5%)		
Physical Activity (*n* = 203)	Active	11 (5.4%)	95 (46.8%)	0.168	–
	Inactive	5 (2.5%)	92 (45.3%)		
Sedentary Lifestyle (*n* = 203)	Yes	10 (4.9%)	151 (74.4%)	0.084	–
	No	6 (3.0%)	36 (17.7%)		
Aggregate factors^a^ (*n* = 203)	No Factor	4 (2.0%)	15 (7.4%)	0.001	3.0 (0.8–10.9)
	1 Factor	9 (4.4%)	101 (49.8%)		
	2 Factors	3 (1.5%)	71 (35.0%)		6.3 (1.3–31.2)
Active Transportation (*n* = 179)	Yes	3 (1.7%)	61 (34.1%)	0.244	–
	No	11 (6.1%)	104 (58.1%)		
Socioeconomic profile (*n* = 136)	Low	4 (2.0%)	46 (22.7%)	0.312	–
	Medium	6 (3.0%)	72 (35.5%)		
	High	2 (1.0%)	6 (3.0%)		
Triglycerides (*n* = 197)	Normal	15 (7.6%)	1 (0.5%)	0.315	–
	High	148 (75.1%)	33 (16.8%)		
Cholesterol Total (*n* = 197)	Normal	11 (5.6%)	39 (19.8%)	0.000	8.0 (2.6–24.4)
	High	5 (2.5%)	142 (72.1%)		
Blood Glucose (*n* = 193)	Normal	16 (8.3%)	176 (91.2%)	0.763	–
	High	0 (0%)	1 (0.5%)		

^a^Aggregate factors: a) adequate physical activity and not sedentary (classified as “no factor”); b) either no physical activity or sedentary (classified as “1 factor”); c) low levels of physical activity and sedentary (classified as “2 factors”)

The analysis for both genders indicated that individuals with aggregated factors had a direct association with excess fat. Thus, the presence of two factors (physical inactivity and sedentary lifestyle) increases the risk by 11.3 times for boys (95% CI: 2.6–50.2) and 6.3 times for girls (95% CI: 1.3–31.2).

In boys, there was a significant association between excess fat and waist circumference (*p* = 0.000; OR = 13.5), physical activity level (p = 0.000; OR = 4.0), triglycerides (*p* = 0.019; OR = 2.2) and cholesterol (p = 0.000; OR = 2.6). In girls, an association between excess fat and cholesterol (p = 0.000; OR = 8.0) was found.

**Fig. 1:**
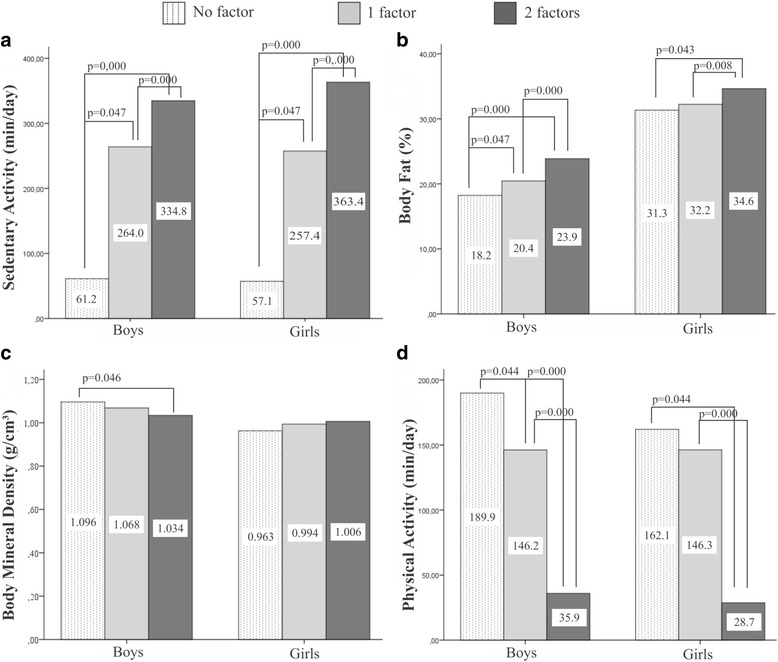
Graphical analysis of the sample stratification according to the existence of aggregated factors and associated variables: time spent with sedentary activities (**a**); fat mass (**b**); bone mineral density (**c**); and length of physical activity (**d**). Legend: Where: adequate physical activity and not sedentary (classified as “no factor”); either no physical activity or sedentary (classified as “1 factor”); low levels of physical activity and sedentary (classified as “2 factors”)

Figure 1 presents the results of the graphical analysis of the group stratification according to the existence of aggregated factors (No factor, 1 factor, 2 factors) and associated variables: time spent with sedentary activities (a), fat mass (b), bone mineral density (c) and length of physical activity (d).

It was verified that adolescents with no risk factors associated to their life habits spend more time with physical activities and less time with sedentary activities when compared to those with 1 or 2 factors. In addition to that, BMD values were lower in boys with 2 aggregated factors when compared to those with no factor (*p* = 0.046).

## Discussion

### Prevalence of excess fat

The metropolitan area of Curitiba is the eighth most populous area in Brazil. The HDI (Human Development Index) of Curitiba is 0.823, considered to be very high, similar to some developed countries such as Poland (0.853). However, it is the sixth most violent capital in the country. As most of the southern Brazilian population, Curitiba is inhabited mostly by Brazilians who are descendant from European cultures, especially Polish, Ukrainian, Italian, German and Asian.

In our study, it was detected a critical stage in the prevalence of excess fat (40.4%), higher than the projection for the year 2025 [9]. In addition to it, this prevalence was much higher among female adolescents (92.1%) than males (18.2%). In concordance with our results, another study using DXA, performed in the southeast region of Brazil, with 215 adolescents aged 10 to 14 years, found a prevalence of 44.2% of excess fat, without stratification by sex [19]. The high prevalence of overweight and obesity, as high as 38.9%, was also indicated by a review study composed of 16 researches made in Brazil [7]. This review study, as well as ours, shows that Brazilian public policies focused on low weight might be equivocal. Thus, differently from previous decades when undernutrition was a chronic problem in our country, the new reality seems to point towards high prevalence of overweight and obesity. Therefore, this new information should be considered both when evaluating the maintenance of existing public policies and when creating new control policies for overweight and obesity in adolescence.

The percentage of fat mass found in our study was 24.5 ± 8.0% (20.9 ± 5.9% for boys and 33.0 ± 5.4% for girls). These are so high that come close to numbers found in the USA, as shown in a study conducted among USA children and adolescents, those being 25.4 ± 0.2% for boys and 33.3 ± 0.3% for girls [20]. On the other hand, a study conducted in Poland, a developed country with an HDI similar to the area of our study, found a body fat 15.6% in boys and 13.4% in girls, all aged between 14 and 18 years [21].

Nevertheless, in Asian populations lower values of fat mass were found, such as in a study among adolescents of Malay and Chinese origins (aged 12 to 19). For boys, values of 17.1 ± 10.0% and 18.8 ± 9.4% were found for Malays and Chinese, respectively. For girls, the values found were 31.7 ± 8.4% and 32.8 ± 7.1% for Malays and Chinese, respectively [22]. Although these values are smaller than ours, this study also points to greater fat mass values in girls.

### Physical activity and sedentary behavior

Regarding the relationship between physical inactivity, gender and obesity, some studies have also found association between these variables. In our study, male adolescents were more active than females (66.3% and 52.2%, respectively). In sum, the inactive had 2.9 times more chance of being classified with excess fat (4.0 times more among boys). Female adolescents had more sedentary habits (79.3%) when compared to boys (75.2%). This could be a factor in explaining the higher prevalence of excess fat among girls, since the act of watching television has been related to the consumption of high-calorie food [7, 8].

In comparison to the behavior of boys, more frequent sedentary behavior among girls has been reported in other studies conducted in Brazil and in Asian countries. In Goiânia, Brazil, a study with adolescents aged 14 to 18 years found that 78% of the girls and 54.3% of the boys were sedentary [11]. A study in Malaysia with adolescents with ages ranging from 12 to 19 years (Malay and Chinese origin) showed a significant relationship between excess fat and sedentary behavior only among girls. Separating by ethnicity, girls of Malay origin had sedentary behavior on average of 3.1 h/day while those of Chinese origin reached 3.8 h/day [23].

Another Brazilian study carried out in São Paulo, with children and adolescents (aged 7 to 18), found that 28.3% of the boys and 20.4% of the girls were overweight/obese. Among the risk factors were: going to school by car (0.72) (when compared to going by bus 0.61 and walking 0.59), more than one hour of computer use per day (1.64) or more than two hours per day (1.94) [6].

In Brazil, full-time schools are rare. Therefore, the time available for performing sedentary activities (use of television, computer, and video game) is higher than in developed countries [7].

### Active transportation and socio-economic status

We did not find relation between the excess fat in adolescents with the income or the way of transportation to the school (active transportation). Active transportation is adopted by a small fraction of the adolescents in the area of the study [24]. Violence might be an impacting factor, for active transportation was more common among boys (19.4% than girls (6.4%). When investigating the causes for this low rate, 84% of the parents and/or guardians show concern as to attacks of strangers being a barrier to adopting active transportation.

A study carried out in seven African developing countries meets our results. It showed that the active transportation to school did not influence the risk of overweight/obesity in adolescents in Benin, Djibouti, Egypt, Malawi and Mauritania. However, it was found that in Morocco the active transportation appeared to reduce the odds (OR = 0.77; 95% CI: 0.61–0.97) and that the opposite occurred in Ghana, where the odds appeared to have increased (OR = 1.28; 95% CI: 1.01–1.62) [9].

Despite there is no concordance in literature, the majority of the studies have not found significant relations between excessive body fat and risk factors as socio-economic level of the families, education level of the parents, among others [7, 12].

In the southeastern region of Brazil, authors also reported not having found a significant association between the type of school, total annual family income, employment status or maternal or paternal schooling with obesity and levels of physical activity [12].

Conversely, a review study (with 16 articles published between 2004 and 2012 in Brazil) showed that only one study, conducted in the center-west region, indicated some relation between overweight/obesity and socioeconomic level [7].

### Abdominal obesity and biomarkers

The male adolescents who presented abdominal obesity had 13.5% higher risk of being classified with excess fat. This risk was not verified among the girls. Although girls have a higher percentage of body fat due to hormonal differences between the sexes, they tend to accumulate adipose tissue in the hip region, while boys tend to accumulate it in the waist region [8, 25].

A study conducted in São Paulo, Brazil, with adolescents aged 14 to 19, found a prevalence of abdominal obesity of 10.5% in males and 10.8% in females. The authors present sedentary behavior as the main lifestyle factor associated with abdominal obesity [8].

A study conducted in the same region as our study, with adolescents aged 11 to 19, identified a prevalence of general abdominal obesity of 12.2%, which was also higher in boys (15.1%) than in girls (12.2%) [26].

Comparing the prevalent values between developed and developing countries, a review study found that the prevalence of abdominal obesity in developing countries varied from 3.8% to 51.7%. In developed countries, however, rates ranged from 9.3% to 33.2%. Many of these discrepancies may have occurred due to the different criteria applied [27].

The adoption of public policies to restrain the excessive body fat is important because one of its consequences might be the alteration of biochemical parameters. In our study, the prevalence of high total cholesterol was 22.2% in boys and 42.3% in girls. Considering those with excess fat, boys were 2.2 times more likely to have increased triglycerides and 2.67 times more likely to have high total cholesterol. In girls, obesity seems to have affected only total cholesterol. Girls with excess fat had 8.0 times more chance of having high total cholesterol.

### Bone health

Risks for cardiovascular health and metabolic syndrome of the increased rates of cholesterol and triglycerides are well documented. However, some researches are also concerned about their impact on bone health. It seems this relation should be further investigated.

Our study found no direct relationship between excess fat and Bone Mineral Density (BMD). However, BMD values were lower (*p* = 0.046) in boys with two aggregate factors (physical inactivity and sedentary lifestyle). This is worrisome because adolescence is considered a critical period for the gain of bone mass and an impaired bone acquisition during this phase may increase the risk of osteopenia/osteoporosis and fractures in old age [28].

A possible explanation for the effect of HDL cholesterol on the skeleton would be the inhibition of osteogenic tissue, suggesting that it regulates the differentiation of osteoblasts. Therefore, the authors describe a study that detected a negative correlation between hypercholesterolemia with BMD (lumbar spine) and BMC (Bone Mineral Content) and increased total cholesterol in overweight/obese girls, but not in boys. The authors further suggested that the risk of cardiovascular disease and osteoporosis later in life would be greater in these girls [28, 29].

The authors describe another study that negatively correlated the increase in triglyceride levels and BMD in adolescents. It was observed that triglycerides were positively correlated with fat in the bone marrow, possibly because this type of fat stimulates osteoclasts (which degrade bone tissue) and it was also noted that high levels of bone marrow fat increased the risk of fracture [27, 30].

A review study that included 27 studies described that overweight or obese children have a significantly higher BMD than normal-weight children. That sensitivity analysis showed that the association was stronger in girls [31]. However, the authors found only one longitudinal study that investigated the long-term consequences of obesity in BMD. This longitudinal study found a higher trabecular density at the tibia in adult women and a lower cortical density at the tibia in adult men who were obese in childhood. In addition to these findings, the authors describe that the results of few studies that compared sexes indicate significant differences between normal weight, overweight or obesity are found more often in girls than in boys. This can occur due to the difference in hormonal development in girls and boys. Considering this result and the lack of evidence on the long-term consequences of childhood obesity in BMD, further prospective research is recommended [31].

It is worth noticing that bone quality in obesity is not as good as that obtained by physical activity. Some authors have related resistance to leptin, frequently found in obese children, due to poor bone health. They suggest that leptin has an indirect effect on bone formation influencing other hormones that affect bone density such as growth hormone, androgens, and cortisol. Thus, the microstructure of the bone would be altered and this would lead to a greater propensity to fractures, especially of the forearm, in obese children [31, 32].

### Strengths and limitations

Among the limitations of this research is the transversal design, offering no basis for the study of probabilities. Another limitation was the use of questionnaires to collect data regarding sedentary behavior, active transportation and level of physical activity. Although validated, the instruments may contain a bias of estimation and memory. Moreover, the analyzed adolescents are from a single region of the country, not providing a nationally representative sample.

More than 3000 adolescents were contacted, but we still had great difficulty in sensitizing them to participate in the research. Therefore, we analyzed data from adolescents who either had not filled any questionnaire or not agreed to take any specific collection (such as blood testing). This way, another limitation in this study was a lot of missing values, for example, socioeconomic data. The use of a single body fat cut-off to define obesity (25% in boys and 30% in girls) is also mentioned as limitation. The use of age-specific cut-offs may be more suitable to define pediatric obesity.

Another limitation was not being able to investigate the adolescents’ diets. Besides physical activity, diet is one of the most important determinants of adiposity. In this sense, high prevalence of excessive body fat might have been influenced by an hypercaloric and unhealthy diet.

Despite these limitations, this study is important because it uses DXA-based %BF to identify overweight and obese adolescents. In addition, it was possible to obtain objective and consistent measures of several risk factors that may help in the elaboration of local public policies, since Brazil is a continental country with great ethnic and cultural variety among its regions. Thus, it is possible to establish local strategies to help fight overweight/obesity among adolescents in this particular region.

Moreover, the use of DXA provided us with important information about BMD, justifying future studies investigating the relationships between BMD and excess fat, levels of physical activity, and rates of cholesterol and triglycerides.

## Conclusion

The high prevalence of obesity found (40.4%) was higher than that described by some studies carried out in developed countries, demonstrating the hypothesis that excess fat is already a serious public health problem in the studied region.

In this study, the risk factors for boys were mainly related to the low level of physical activity. The impacts could be observed in the greater waist circumference, in increased levels of total cholesterol, triglycerides, and in lower bone mineral density.

For the girls, the risk factors had some aggregated risk factor for physical inactivity and/or sedentary lifestyle. Consequently, as in boys, it was possible to visualize the increase in total cholesterol levels.

Based on these results, we suggest a re-evaluation of the public policies which are focused on undernutrition, since the epidemiologic pattern seems to be being altered to a high prevalence of overweight and obesity among adolescents. Furthermore, we recommend the adoption of a program to monitor serum levels of cholesterol and triglycerides. In Brazil, during infancy, children regularly attend the pediatrician, due to the need for vaccination. However, in adolescence, this monitoring no longer happens. Thus, this picture may be going unnoticed.

Finally, this serious reality demonstrates the need to adopt public policies that may directly influence the reduction of sedentary habits and reinforce the importance of keeping an active lifestyle, especially for girls.

## Additional files

Additional file 1: Definition of the statistically significant sample size. (DOCX 13 kb)

Additional file 2: Descriptive analysis of data derived from excess fat, sedentary lifestyle, physical activity and biochemical markers in different age groups. (DOCX 22 kb)

## Abbreviations

BMD: Bone Mineral Density
BMI: Body Mass Index
CI: Confidence Interval
TC: Total Cholesterol
DXA: Dual Energy X-ray Absorptiometry
MetS: Metabolic Syndrome
OR: Odds Ratio
WC: Waist Circumference
