# Supplementary material for: Prevalence of excessive body fat among adolescents of a south Brazilian metropolitan region and State capital, associated risk factors, and consequences
Source: BMC Public Health. 2018 Mar 2;18:312. doi: 10.1186/s12889-018-5216-0 (PMC5834854; doi:10.1186/s12889-018-5216-0)
Supplement: Supplementary file 1 — Definition of the statistically significant sample size. (DOCX 13 kb) [file 12889_2018_5216_MOESM1_ESM.docx]

**SUPPORTING INFORMATION #1**

For the definition of the statistically significant sample size, a minimum of 354 people was calculated. For this, we used the equation (1) defined by Cochran [1], where a sample error of 4.5% was specified under the 91% confidence level based on a population of 82,363 individuals obtained through the national Brazilian database system (Datasus) [2] of 2011.

$n= \frac{N \times Z^{2} \times p (1-p)}{Z^{2} \times p \left( 1-p \right)+ e^{2} (N-1)}$ (1)

Where:

$n$ = sample;

N= population;

Z = standardized normal variable associated with the confidence level;

P = true probability of the event;

e = sampling error.

REFERENCES:

[1] COCHRAN, William G. **Sampling Techniques**. 3a:John Wiley & Sons, 1977.

[2] DATASUS, <http://datasus.saude.gov.br/>,
