# Supplementary material for: Prevalence of excessive body fat among adolescents of a south Brazilian metropolitan region and State capital, associated risk factors, and consequences
Source: BMC Public Health. 2018 Mar 2;18:312. doi: 10.1186/s12889-018-5216-0 (PMC5834854; doi:10.1186/s12889-018-5216-0)
Supplement: Supplementary file 2 — Descriptive analysis of data derived from excess fat, sedentary lifestyle, physical activity and biochemical markers in different age groups. (DOCX 22 kb) [file 12889_2018_5216_MOESM2_ESM.docx]

**SUPPORTING INFORMATION #2**

In Table 5 and 6 there is an additional analysis concerning the prevalence on excess fat, biomarkers, waist circumference, socioeconomic data, sedentary lifestyle, physical activity, and active transportation in different age groups. Data were subdivided into boys (Table 5) and girls (Table 6).

Table 5. Descriptive analysis of data derived from excess fat, sedentary lifestyle, physical activity and biochemical markers in boys.

| **Age Group** | **11 - 13** | **14 - 15** | **16 - 18** | **All** | **p** |
| --- | --- | --- | --- | --- | --- |
| N | 119 | 181 | 172 | 472 |  |
| Weight (kg) | 49.5±12.4*† | 60.5±10.9† | 66.5±12.5 | 59.9±13.5 | 0.000 |
| Height (m) | 1.56±0.09*† | 1.69±0.07† | 1.74±0.1 | 1.67±0.1 | 0.000 |
| BMI (kg/m²) | 20.2±4.0† | 21.06±3.02† | 22.2±5.3 | 21.3±4.3 | 0.000 |
| WC (cm) | 68.0±9.1† | 70.3±7.8† | 73.6±8.3 | 70.9±8.6 | 0.000 |
| Fat Mass (%) | 25.2±6.6*† | 19.9±4.8† | 18.8±4.8 | 20.9±5.9 | 0.000 |
| Bone Mineral Density (g/cm²) | 0.913±0.08*† | 1.061±0.111† | 1.151±0.109 | 1.064±0.137 | 0.000 |
| *%BF Classification* |  |  |  |  |  |
| Normal | 69 (58.0%) | 161 (89.0%) | 156 (90.7%) | 386 (81.8%) | - |
| High | 50 (42.0%) | 20 (11.0%) | 16 (9.3%) | 86 (18.2%) | - |
| *BMI Classification* |  |  |  |  |  |
| Normal Weight | 74 (62.2%) | 123 (68.0%) | 104 (60.5%) | 330 (69.9%) | - |
| Overweight / Obesity | 45 (37.8%) | 57 (31.5%) | 67 (39.0%) | 142 (30.1%) | - |
| *Biochemical Classification* |  |  |  |  |  |
| Cholesterol Normal | 46 (38.7%) | 91 (50.3%) | 87 (50.6%) | 224 (47.5%) | - |
| Cholesterol Limit (150-169 mg/dl) | 25 (21.0%) | 41 (22.7%) | 52 (30.2%) | 118 (25.0%) | - |
| Cholesterol High (≥170 mg/dl) | 35 (29.4%) | 37 (20.4%) | 33 (19.2%) | 105 (22.2%) | - |
| Cholesterol (unrealized) | 13 (10.9%) | 12 (6.6%) | 0 (0%) | 25 (5.3%) | - |
| Blood Glucose Normal | 107 (89.9%) | 168 (92.8%) | 170 (98.8%) | 445 (94.3%) | - |
| Blood Glucose High (≥140mg/dl) | 0 (0%) | 2 (1.1%) | 2 (1.2%) | 4 (0.8%) | - |
| Blood Glucose (unrealized) | 12 (10.1%) | 11 (6.1%) | 0 (0%) | 23 (4.9%) | - |
| Triglycerides Normal | 92 (77.3%) | 151 (83.4%) | 152 (88.4%) | 395 (83.7%) | - |
| Triglycerides High (≥150mg/dl) | 12 (10.1%) | 19 (10.5%) | 19 (11.0%) | 50 (10.6%) | - |
| Triglycerides (unrealized) | 15 (12.6%) | 11 (6.1%) | 1 (0.6%) | 27 (5.7%) | - |
| *Physical activity level* |  |  |  |  |  |
| Time (hours/day) | 1.4±2.0*† | 2.5±2.2 | 2.3±1.7 | 2.2±2.0 | 0.000 |
| Active | 50 (42.0%) | 131 (72.4%) | 132 (76.7%) | 313 (66.3%) | - |
| Inactive | 69 (58.0%) | 50 (27.6%) | 40 (23.3%) | 159 (33.7%) | - |
| *Sedentary lifestyle* |  |  |  |  |  |
| Time in week (hours/day) | 3.4±3.6*† | 4.5±3.6 | 4.8±3.1 | 4.3±3.5 | 0.003 |
| Time in weekend (hours/day) | 7.2±6.4*† | 6.8±5.5 | 6.5±5.9 | 6.5±5.9 | 0.004 |
| % of sedentary | 69 (58.0%) | 136 (75.1%) | 150 (87.2%) | 355 (75.2%) | - |
| *Socioeconomic profile* |  |  |  |  |  |
| High | 4 (3.4%) | 8 (4.4%) | 9 (5.2%) | 21 (4.4%) | - |
| Medium | 38 (31.9%) | 64 (35.4%) | 68 (39.5%) | 170 (36.0%) | - |
| Low | 55 (46.2%) | 69 (38.1%) | 70 (40.7%) | 194 (41.1%) | - |
| Not answered | 22 (18.5%) | 40 (22.1%) | 25 (14.5%) | 87 (18.5%) | - |
| *Active transportation* |  |  |  |  |  |
| Yes | 47 (39.5%) | 61 (33.7%) | 46 (26.7%) | 154 (32.6%) | - |
| No | 38 (31.9%) | 61 (33.7%) | 75 (43.6%) | 174 (36.8%) | - |
| Not answered | 34 (28.6%) | 59 (32.6%) | 51 (29.7%) | 144 (30.6%) | - |

Where: waist circumference (WC); body mass index (BMI); fat percentage (%BF); physical activity (PA); significative difference between age group 14-15 (*); significative difference between age group 16-18 (†);

The analysis for boys indicated that younger individuals (11-13 years) had significantly less values in all anthropometric variables. In addition, this group spent less time (hours/day) in physical activity and sedentary lifestyle.

Table 6. Descriptive analysis of data derived from excess fat, sedentary lifestyle, physical activity and biochemical markers in girls.

| **Age Group** | **11 - 13** | **14 - 15** | **16 - 18** | **All** | **p** |
| --- | --- | --- | --- | --- | --- |
| N | 70 | 55 | 78 | 203 |  |
| Weight (kg) | 50.6±10.2*† | 56.2±10.7 | 60.5±11.3 | 55.9±11.5 | 0.000 |
| Height (m) | 1.56±0.1*† | 1.61±0.1 | 1.61±0.1 | 1.59±0.1 | 0.000 |
| BMI (kg/m²) | 21.0±3.4† | 22.2±4.5 | 23.4±3.9 | 22.3±4.06 | 0.001 |
| WC (cm) | 66.7±7.1† | 68.6±7.4 | 70.9±8.4 | 68.8±7.9 | 0.005 |
| Fat Mass (%) | 32.0±5.6† | 32.5±5.3 | 34.3±5.2 | 33.0±5.4 | 0.025 |
| Bone Mineral Density (g/cm²) | 0.906±0.074*† | 1.002±0.072† | 1.064±0.109 | 0.995±0.112 | 0.000 |
| *%BF Classification* |  |  |  |  |  |
| Normal | 8 (11.4%) | 4 (7.3%) | 4 (5.1%) | 16 (7.9%) | - |
| High | 62 (88.6%) | 41 (92.7%) | 74 (94.9%) | 187 (92.1%) | - |
| *BMI Classification* |  |  |  |  |  |
| Normal Weight | 47 (67.1%) | 41 (74.5%) | 61 (78.2%) | 121 (59.6%) | - |
| Overweight / Obesity | 23 (32.9%) | 14 (25.5%) | 17 (21.8%) | 82 (40.4%) | - |
| *Biochemical Classification* |  |  |  |  |  |
| Cholesterol Normal | 23 (32.9%) | 15 (27.3%) | 12 (15.4%) | 50 (24.6%) | - |
| Cholesterol Limit (150-169 mg/dl) | 20 (28.6%) | 17 (30.9%) | 24 (30.8%) | 61 (30.0%) | - |
| Cholesterol High (≥170 mg/dl) | 26 (37.1%) | 19 (34.5%) | 41 (52.5%) | 86 (42.3%) | - |
| Cholesterol (unrealized) | 1 (1.4%) | 4 (7.3%) | 1 (1.3%) | 6 (3.0%) | - |
| Blood Glucose Normal | 63 (90.0%) | 51 (92.7%) | 78 (100%) | 192 (94.6%) | - |
| Blood Glucose High (≥140mg/dl) | 0 (0%) | 1 (1.8%) | 0 (0%) | 1 (0.5%) | - |
| Blood Glucose (unrealized) | 7 (10%) | 3 (5.5%) | 0 (0%) | 10 (4.9%) | - |
| Triglycerides Normal | 51 (72.9%) | 46 (83.6%) | 66 (84.6%) | 163 (80.3%) | - |
| Triglycerides High (≥150mg/dl) | 17 (24.3%) | 6 (10.9%) | 11 (14.1%) | 34 (16.7%) | - |
| Triglycerides (unrealized) | 2 (2.8%) | 3 (5.5%) | 1 (1.3%) | 6 (3.0%) | - |
| *Physical activity level* |  |  |  |  |  |
| Time (hours/day) | 1.9 | 1.9 | 1.5 | 1.7±2.1 | 0.405 |
| Active | 40 (57.1%) | 35 (63.6%) | 31 (39.7%) | 106 (52.2%) | - |
| Inactive | 30 (42.9%) | 20 (36.4%) | 47 (60.3%) | 97(47.8%) | - |
| *Sedentary lifestyle* |  |  |  |  |  |
| Time in week (hours/day) | 4.5±3.8 | 4.7±3.6 | 4.7±2.9 | 4.6±3.4 | 0.956 |
| Time in weekend (hours/day) | 6.0±5.5 | 5.6±4.3 | 6.1±4.0 | 5.9±4.6 | 0.808 |
| % of sedentary | 50 (71.4%) | 43 (78.2%) | 68 (87.2%) | 161 (79.3%) | - |
| *Socioeconomic profile* |  |  |  |  |  |
| High | 3 (4.3%) | 2 (3.6%) | 3 (3.8%) | 8 (3.9%) | - |
| Medium | 29 (41.5%) | 16 (29.1%) | 33 (42.3%) | 78 (38.4%) | - |
| Low | 19 (27.1%) | 15 (27.3%) | 16 (20.5%) | 50 (24.6%) | - |
| Not answered | 19 (27.1%) | 22 (40.0%) | 26 (33.4%) | 67 (33.1%) | - |
| *Active transportation* |  |  |  |  |  |
| Yes | 28 (40.0%) | 18 (32.7%) | 18 (23.1%) | 64 (31.6%) | - |
| No | 31 (44.3%) | 28 (50.9%) | 56 (71.8%) | 115 (56.6%) | - |
| Not answered | 11 (15.7%) | 9 (16.4%) | 4 (5.1%) | 24 (11.8%) | - |

Where: waist circumference (WC); body mass index (BMI); fat percentage (%BF); physical activity (PA); significative difference between age group 14-15 (*); significative difference between age group 16-18 (†);

The analysis for girls indicated no difference in physical activity levels and time with sedentary habits in any group. As expected, the age group (11-13 years) shows less weight, height, body fat and BMD.
